# Enhancing Radiology Reporting Standards: A Two-Cycle Audit Based on the Royal College of Radiologists (RCR) 2018 Guidelines in a Tertiary Gastroenterology and Hepatology Center in Iraq

**DOI:** 10.7759/cureus.85339

**Published:** 2025-06-04

**Authors:** Yousif Alhabar, Talal Non, Asama Ijam

**Affiliations:** 1 Internal Medicine, Basrah Gastroenterology and Hepatology Hospital, Basrah, IRQ; 2 Gastroenterology and Hepatology, Basrah Gastroenterology and Hepatology Hospital, Basrah, IRQ; 3 Interventional Radiology, Al-Moosawi Private Hospital, Basrah, IRQ

**Keywords:** actionable reporting, benchmarking standards, clinical audit, quality improvement, radiological imaging, radiology reporting, rcr standards, structured reporting

## Abstract

Background

High-quality radiology reports are crucial for informing clinical decisions and ensuring patient safety, particularly in a tertiary center like Basra Gastroenterology and Hepatology Hospital (BGHH), Iraq. However, at our institution, many reports lacked key elements such as actionable recommendations, provisional diagnoses, and differential diagnoses, which are essential for clinician-focused reporting. To address these deficiencies, we conducted a two-cycle audit, benchmarking our practice against the 2018 Royal College of Radiologists (RCR) “Standards for Interpretation and Reporting of Imaging Investigations".

Objective

This audit aims to assess whether radiological reports produced at BGHH adhered to the 2018 RCR standards for actionable reporting and to evaluate whether targeted interventions could improve compliance.

Methodology

A two-cycle clinical audit was conducted. During the first cycle in January 2025, 100 radiology reports were randomly selected and analyzed against RCR standards. After identifying key gaps, four targeted interventions were introduced, including departmental training and a structured reporting format. In the second cycle (March-April 2025), another 100 reports were reviewed to evaluate the impact of these changes.

Results

After implementing the interventions, we observed a marked improvement in report quality and compliance with RCR standards. Actionable reporting reached 100%, compared to 95% in the initial cycle (p < 0.05), eliminating any reports lacking clear clinical direction. The presence of advice for next steps, a critical component of actionable reporting, improved dramatically from 13% to 100%, ensuring all reports offered guidance for further management. Furthermore, the presence of differential diagnoses rose from 11% to 94% (p < 0.05), reflecting enhanced clinical reasoning within reports. These improvements demonstrate the effectiveness of structured training and standardized templates in elevating the consistency and clinical utility of radiology reporting.

Conclusion

This audit shows that implementing simple, focused changes led to measurable improvements in the clarity, structure, and clinical usefulness of radiology reports at BGHH. These findings show that even low-cost interventions can enhance report quality and align practice with international standards, ultimately supporting better clinical decision-making. This audit also provides a practical template that could be replicated across other hospitals in Iraq to promote nationwide improvement in radiology reporting.

## Introduction

Radiology reports play a pivotal role in modern healthcare by delivering diagnostic information that significantly influences clinical decision-making. The quality of these reports directly impacts patient outcomes, making accuracy, clarity, and consistency essential components of effective radiological communication [1]. In recognition of this, the Royal College of Radiologists (RCR) published the second edition of its Standards for Interpretation and Reporting of Imaging Investigations in 2018 [2]. These guidelines emphasize the importance of “actionable reporting,” defined as reports that clearly address the clinical question, highlight relevant findings, and offer guidance on further management steps.

Actionable reporting is more than just a professional expectation; it is a cornerstone of safe and effective patient care. Inadequate reports that lack clear clinical context, relevant findings, or management recommendations can delay diagnosis and treatment, potentially leading to adverse outcomes. Boland et al. [3] and Young et al. [4] highlight the value of comprehensive reporting, noting that reports that include differential diagnoses and follow-up recommendations are more likely to result in appropriate clinical actions. Consistency in reporting not only facilitates timely communication between radiologists and referring clinicians but also helps prevent misinterpretation and miscommunication [5].

Despite the clear guidance provided by the RCR, challenges to consistent implementation persist. Factors such as time pressure, increasing workload, and variable institutional practices may lead to variability in report quality and adherence to established standards. To overcome these barriers, targeted strategies such as structured reporting templates and focused training initiatives have been recommended to support radiologists in producing reports that meet high standards of quality and clinical relevance [6].

In Iraq, however, there is a notable lack of published data evaluating radiology report quality against international benchmarks. This audit was therefore conducted at a major tertiary referral center in Iraq to assess current reporting practices and identify areas for improvement. The goal was to evaluate compliance with the RCR 2018 standards and promote actionable reporting as a means of enhancing diagnostic accuracy and patient safety.

## Materials and methods

This audit was conducted in the Department of Radiology at Basrah Gastroenterology and Hepatology Hospital (BGHH), a tertiary care center in southern Iraq. The project was designed to evaluate adherence to the Standards for the Interpretation and Reporting of Imaging Investigations published by the RCR in 2018 [2]. These standards emphasize the need for clear, consistent, and actionable radiology reporting.

In compliance with local regulations from the Iraqi Ministry of Health, formal ethical approval is not required for clinical audits. Nevertheless, the audit protocol received approval from both the Radiology Department and the Clinical Governance Department at BGHH December 17, 2024 to ensure it aligned with institutional ethical practices.

Sample determination

Based on guidance from the RCR recommendation, a minimum of 100 reports was required to ensure adequate statistical power for audit-based analysis [7]. Therefore, we selected a sample size of 100 radiology reports for each audit cycle. This number was considered sufficient to detect meaningful differences in reporting quality before and after the intervention while also remaining feasible within the constraints of the audit period.

Sampling technique

For both audit cycles, we applied a computer-generated random number table to select 100 reports from all imaging studies performed within the relevant audit periods. Cycle One included reports from January 2025, and Cycle Two covered the period between March and April 2025. Exclusion criteria were applied to omit any reports that described only normal findings or findings unrelated to the clinical question posed by the referring clinician. For each included report, we documented patient demographic data and the clinical indication for the imaging.

Audit standards

The audit was structured around key components of the 2018 RCR standards. Table 1 provides a summary of the 16 primary reporting standards evaluated, including expectations related to clinical relevance, communication of findings, documentation practices, feedback mechanisms, and professional accountability. For the purpose of this audit, we focused on three core elements: whether the report answered the clinical question, whether it included a diagnosis or differential when abnormalities were found, and whether any advice provided was appropriate and relevant to the clinical context. These elements were selected because they are central to the concept of actionable reporting, and the recommendation is a 100% compliance target for each [7].

**Table 1:** The 2018 RCR Reporting Standards Summary RCR: Royal College of Radiologists; RIS: Radiology Information Systems; PACS: Picture Archiving and Communication Systems; MDTM: multidisciplinary team meetings; FRCR: Fellow of the Royal College of Radiologists; LDMs: learning from discrepancy meetings; CPD: continuing professional development; GMC: General Medical Council

No.	RCR Standard
1	Reports should be actionable, answer the clinical question, and include a diagnosis or differential diagnosis with relevant negative findings.
2	Report wording should be clear and tailored to the referrer’s background. Recommendations for further action should be included when relevant.
3	Reporters should review prior imaging, clinical data, lab results, and histopathology when available.
4	Long reports should end with a summary of key findings and clinical advice if appropriate.
5	All reports must be formally documented in RIS-PACS and visible in the electronic patient record (EPR).
6	Medical emergencies or unexpected findings must be escalated via local alerting protocols.
7	Supplementary verbal communication is encouraged when appropriate; discussion pathways must exist.
8	Second opinions and ad hoc reviews must be formally recorded as supplementary reports and highlighted to referrers if clinical interpretation changes.
9	Reporters should receive structured feedback (e.g., via MDTMs) as part of their professional practice.
10	All reporters should engage in quality assurance activities like LDMs and peer feedback.
11	Reporters must meet national standards (e.g., FRCR), CPD, annual appraisal, and five-year revalidation.
12	All reporters must be licensed and registered with the appropriate regulatory body (e.g., GMC).
13	The reporter’s professional and registration details should be visible on every report.
14	The signing individual is responsible and accountable for the report’s content.
15	Teamwork is essential. Non-medical reporters must have access to medical advice. Radiologists should also have access to second opinions.
16	Reporters must only report within their defined scope of practice and competence.

Cycle One: baseline audit

During the initial audit cycle, referred to as Cycle One, we retrospectively reviewed the selected reports to evaluate compliance with predefined RCR standards. Each report was assessed to determine whether the clinical question was addressed, whether a diagnosis or differential diagnosis was provided for abnormal findings, and whether any recommendations were appropriate to the clinical context. As patient demographics and clinical indications had already been recorded during sampling, the review focused solely on the quality and completeness of the radiology reports.

Intervention phase

After analyzing the baseline findings, the audit team, which consisted of a senior house officer, a consultant radiologist, and a consultant gastroenterologist, convened to design and implement improvement strategies. We introduced four main interventions. First, the team presented the audit results during a formal meeting with departmental radiologists and the head of radiology to highlight deficiencies and discuss corrective actions. Second, the department formally adopted the 2018 RCR standards as the reference framework for all future radiology reporting. Third, a revised reporting structure was introduced, incorporating a standardized template aligned with RCR guidelines (see Appendix). Finally, the head of the radiology department launched a series of monthly educational sessions over a three-month period, focusing on the principles of actionable reporting. These sessions were supplemented by reinforcement of the standards in regular departmental meetings and case-based discussions.

Cycle Two: post-intervention re-audit

To assess the impact of these interventions, a second audit cycle (Cycle Two) was conducted between March and April 2025. Using the same selection criteria and methodology as Cycle One, we randomly reviewed a new sample of 100 radiology reports. The aim was to determine whether the implemented strategies had led to measurable improvements in compliance with the selected RCR standards.

Data analysis

Data analysis was performed using IBM SPSS Statistics for Windows, Version 29.0.2.0 (Released 2021; IBM Corp., Armonk, New York, United States). Descriptive statistics, including mean, standard deviation (SD), percentage, and 95% confidence interval (CI), were used to summarize the findings. To evaluate statistical significance between the two audit cycles, the Chi-square test was applied, with a p-value of <0.05 considered statistically significant.

## Results

Patient demographics

We analyzed a total of 200 radiology reports in this audit, with 100 reports evaluated in each of the two audit cycles. In Cycle One, the mean age of patients was 54.21 ± 11.59 years, with a range of 26-85 years. Female patients represented the majority of cases (66%), while male patients accounted for 34%. In Cycle Two, the mean age slightly increased to 55.86 ± 9.32 years, ranging from 32 to 81 years. Female patients continued to predominate (68%), with male patients comprising 32% of the sample. When combining data from both cycles, the overall average age was 55.04 ± 10.53 years, with female patients accounting for 67% of the total sample.

In terms of patient setting, outpatients constituted 64% of the reports in Cycle One and 69% in Cycle Two. Inpatients represented 36% and 31%, respectively. Overall, outpatients comprised 66.5% of the combined sample, and inpatients 33.5%. A detailed summary of the patient demographics is provided in Table 2.

**Table 2:** Patient demographics The data is presented as n (%), unless otherwise indicated.

Variable/Cycle	Cycle One	Cycle Two		Total
Number of samples	100 (100%)	100 (100%)	200 (100%)	
male	34 (34%)	32 (32%)	66 (33%)	
Female	66 (66%)	68 (68%)	134 (67%)	
Average age (years), mean±SD	54.21±11.59	55.86±9.32	55.04±10.53	
Inpatients	36 (36%)	31 (31%)	67 (33.5%)	
Outpatients	64 (64%)	69 (69%)	133 (66.5%)	

Imaging modalities

We included a total of six different imaging modalities in this audit: abdominal ultrasound, pelvic ultrasound, chest X-ray, barium swallow, computed tomography (CT) of the abdomen, and magnetic resonance cholangiopancreatography (MRCP). Abdominal ultrasound was the most frequently performed imaging modality, accounting for 61% of reports in Cycle One and 70% in Cycle Two. MRCP was the second most common modality, representing 16% and 15% of reports in the two respective cycles. Pelvic ultrasound had the lowest usage in Cycle One (3%), while chest X-ray was the least utilized in Cycle Two (1%). The distribution of imaging modalities across both audit cycles is summarized in Table 3.

**Table 3:** Distribution of imaging modalities The data is presented as n (%). CT: computed tomography; MRCP: magnetic resonance cholangiopancreatography

Imaging Modality	Cycle One	Cycle Two		Total
Pelvic ultrasound	3 (3%)	2 (2%)	5 (2.5%)	
Abdominal ultrasound	61 (61%)	70 (70%)	131 (65.5%)	
Chest X-ray	4 (4%)	1 (1%)	5 (2.5%)	
Barium swallow	4 (4%)	3 (3%)	7 (3.5%)	
CT abdomen	12 (12%)	9 (9%)	21 (10.5%)	
MRCP	16 (16%)	15 (15%)	31 (15.5%)	
Total number	100 (100%)	100 (100%)	200 (100%)	

Comparison of audit cycles

Following the implementation of the targeted interventions, Cycle Two demonstrated statistically significant improvements in multiple report components. Differential diagnoses increased markedly from 11% to 94% (p < 0.05), reflecting a substantial enhancement in clinical reasoning within reports. Advice for the next step rose from 13% to 82% (p < 0.05), ensuring that clinicians received clearer guidance for patient management. Reports providing a definitive answer to the clinical question improved from 96% to 100% (p < 0.05), confirming that nearly all reports directly addressed the referring clinician’s needs. Impressions were present in only 37% of Cycle One reports versus 100% in Cycle Two (p < 0.05), indicating a dramatic shift toward standardized interpretation. Similarly, conclusions or summaries rose from 18% to 100% (p < 0.05), and actionable recommendations increased from 95% to 100% (p < 0.05), underscoring both the comprehensiveness and usability of post-intervention reports.

Several parameters showed consistent high performance across both cycles, with 100% compliance observed in the inclusion of a clinical question, appropriate advice, radiologist identification (name, signature, license, registration), feedback documentation, and access to previous reports and investigations. The full comparison of audit cycle performance is presented in Table 4.

**Table 4:** Comparison of audit cycle performance before and after intervention. Statistical analysis was performed using the Chi-square test, with significance set at p<0.05.

Standard	Cycle One, n (%)	Cycle Two, n (%)		Total, N (%)	P value
Presence of clinical question	100 (100%)	100 (100%)	200 (100%)		—
Presence of an Answer to the clinical question	96 (96%)	100 (100%)	196 (98%)		P<0.05
Presence of provisional diagnosis	94 (94%)	100 (100%)	194 (97%)		P<0.05
Presence of Differential diagnosis	11 (11%)	94 (94%)	105 (52.5%)		P<0.05
Presence of advice for the next step	13 (13%)	82 (82%)	95 (47.5%)		P<0.05
Presence of appropriate advice	100 (100%)	100 (100%)	200 (100%)		—
Presence of actionable recommendation	95 (95%)	100 (100%)	195 (97.50		P<0.05
Presence of impression	37 (37%)	100 (100%)	137 (68.5%)		P<0.05
Presence of conclusion/summary	18 (18%)	100 (100%)	118 (59%)		P<0.05
Presence of clear report language	100 (100%)	100 (100%)	200 (100%)		—
Presence of structured format	99 (99%)	100 (100%)	199 (99.5%)		p>0.05
Presence of access for previous reports	100 (100%)	100 (100%)	200 (100%)		—
Presence of access for previous investigation	100 (100%)	100 (100%)	200 (100%)		—
Presence of systematic feedback	100 (100%)	100 (100%)	200 (100%)		—
Presence of radiologist license registration	100 (100%)	100 (100%)	200 (100%)		—
Presence of radiologist name and signature	100 (100%)	100 (100%)	200 (100%)		—

## Discussion

Radiology plays a central role in modern healthcare, directly shaping clinical decisions and guiding treatment across virtually all medical specialties. Radiologists bear significant clinical responsibility to ensure that their imaging reports contribute meaningfully to clinical care. Radiology reporting is inherently collaborative, operating within multidisciplinary teams and guided by established quality standards that assess both individual and departmental performance [8].

The 2018 RCR standards were developed to improve the clarity, coherence, and clinical relevance of radiology reports. These standards prioritize actionable reporting, effective communication, and multidisciplinary integration, all of which are essential components for delivering safe, timely, and effective patient care [2].

In Cycle One of this audit, a substantial number of reports failed to meet key RCR standards. Our baseline compliance was notably lower than that reported by Sharma et al., who achieved 95% adherence [9]. This discrepancy may reflect challenges common in resource-limited settings, such as high reporting workloads, limited standardization, medicolegal concerns, and variable imaging quality. Additionally, image interpretation can be inherently subjective, and the fear of litigation may discourage radiologists from offering definitive conclusions or clinical advice [10,11]. These challenges reflect a broader need for national and institutional strategies to support quality improvement in radiology reporting.

The improvements seen in Cycle Two highlight the effectiveness of structured audit processes in aligning local practices with international standards. Compliance significantly improved across critical areas such as differential diagnosis, clinical advice, and structured summaries. These changes reflect the positive impact of education, structured feedback, and standardized reporting tools in improving radiological reporting [2].

Adherence to international standards

The 2018 RCR standards emphasize that radiology reports must be actionable, address the clinical question, provide diagnostic impressions or differential diagnoses, and include appropriate management recommendations. Our findings are consistent with those of Sharma et al., who reported improved compliance from 95% to 97% after implementing a similar audit and educational intervention [9]. Adhering to such standards not only promotes consistency in reporting but also improves diagnostic accuracy and contributes to better patient outcomes [12].

Replicability and standardization across Iraq

The success of this audit at BGHH demonstrates that meaningful quality improvement can be achieved through structured audit cycles, multidisciplinary collaboration, and the adoption of internationally recognized standards. This model is scalable and could be replicated in radiology departments across Iraq. Nationwide implementation of standardized reporting protocols would help unify diagnostic practices, streamline interdepartmental communication, and elevate the overall quality of patient care. Furthermore, national standardization would support the development of collaborative audits and research across institutions, fostering a culture of transparency, learning, and continuous improvement in the field of radiology.

Limitations

This audit has several limitations that should be acknowledged. First, it was conducted at a single center, BGHH, and involved a relatively small sample size, which may limit the applicability of our findings to other institutions or larger patient populations. Furthermore, the presence of an electronic health record (EHR) system at BGHH greatly facilitated data collection, organization, and analysis; however, many healthcare facilities in Iraq still rely on paper-based documentation. As a result, replicating this audit model in resource-limited settings would likely require significant planning, investment in digital infrastructure, and dedicated staff training to ensure feasibility and consistency.

Another limitation concerns the sustainability of the improvements observed during Cycle Two. It remains uncertain whether these enhancements reflect genuine long-term behavioral changes or are partly influenced by the Hawthorne effect, whereby participants improve performance due to the awareness of being observed. Without continued oversight, periodic audits, or integrated monitoring systems, there is a risk that adherence to standards may decline over time. To support ongoing compliance, it will be necessary to embed these practices into routine clinical workflows through institutional quality assurance programs, structured feedback systems, and continuous professional education.

## Conclusions

This audit reinforces the critical role of structured radiology reporting and adherence to internationally recognized standards such as those established by the RCR in enhancing the clinical value of imaging reports. The improvements observed following targeted interventions highlight the impact of education, standardization, and interdisciplinary collaboration on elevating reporting quality. By implementing similar strategies, healthcare institutions across Iraq can enhance diagnostic accuracy, optimize clinical workflows, and ultimately improve patient outcomes. However, sustained progress will depend on continued investment in digital infrastructure, routine audit cycles, and ongoing training to support long-term adherence to best practices in radiology reporting.

## Figures and Tables

**Figure 1:**
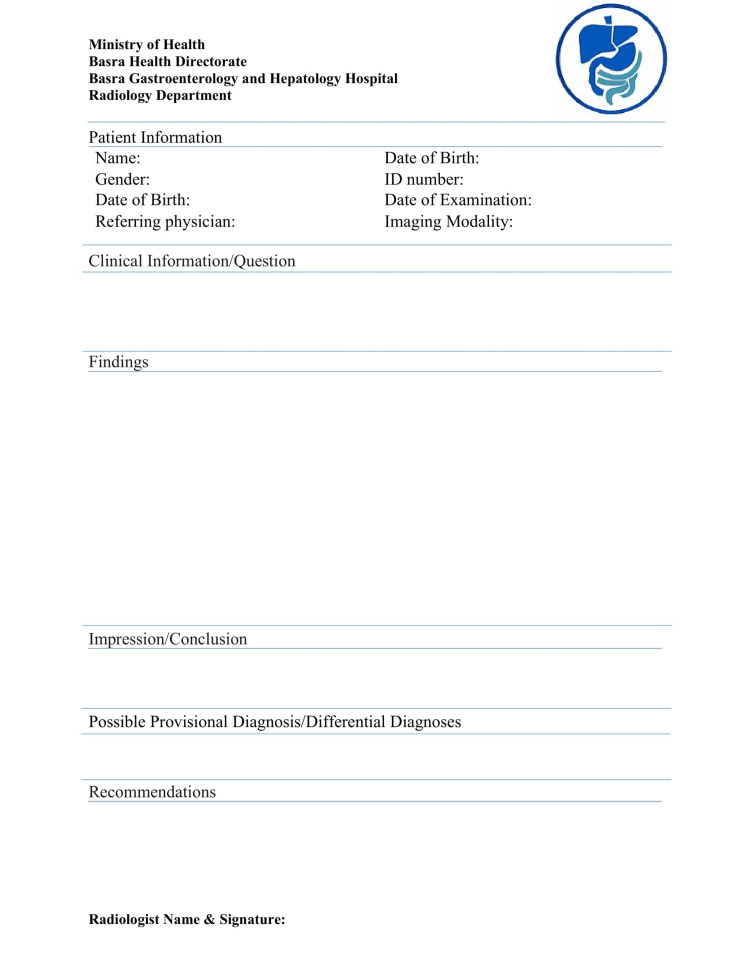
Revised radiology report template aligned with RCR 2018 standards RCR: Royal College of Radiologists

## References

[1] Farmer CI, Bourne AM, O'Connor D, Jarvik JG, Buchbinder R (2020). Enhancing clinician and patient understanding of radiology reports: a scoping review of international guidelines. Insights Imaging.

[2] (2018). Standards for interpretation and reporting of imaging investigations Second edition. Standards for Interpretation and Reporting of Imaging Investigations, Second Edition.

[3] Boland GW, Enzmann DR, Duszak R Jr (2014). Actionable reporting. J Am Coll Radiol.

[4] Young A, Yusuf F, Farthing M, Hafezi-Bakhtiari N (2022). Actionable reporting. Clin Radiol.

[5] Singh H, Giardina TD, Meyer AN, Forjuoh SN, Reis MD, Thomas EJ (2013). Types and origins of diagnostic errors in primary care settings. JAMA Intern Med.

[6] White T, Aronson MD, Sternberg SB (2022). Analysis of radiology report recommendation characteristics and rate of recommended action performance. JAMA Netw Open.

[7] (2025). The Royal College of Radiologists: Actionable reporting [QSI Ref: XR-508]. https://www.rcr.ac.uk/career-development/audit-quality-improvement/auditlive-radiology-templates/actionable-reporting-qsi-ref-xr-508/..

[8] (2022). The role of radiologist in the changing world of healthcare: a white paper of the European Society of Radiology (ESR). Insights Imaging.

[9] Sharma U, Gomindes AR, Sharma K, Choudhry J, C Searle HK (2023). Compliance with the Royal College of Radiologists guideline for actionable reporting and its impact on patient care: a retrospective analysis of reporting practices from a major trauma center. Cureus.

[10] Studdert DM, Mello MM, Sage WM, DesRoches CM, Peugh J, Zapert K, Brennan TA (2005). Defensive medicine among high-risk specialist physicians in a volatile malpractice environment. JAMA.

[11] Sohoni CA (2013). Medical negligence: a difficult challenge for radiology. Indian J Radiol Imaging.

[12] Pesapane F, Tantrige P, De Marco P (2023). Advancements in standardizing radiological reports: a comprehensive review. Medicina (Kaunas).

